# ﻿*Primulinanana* (Gesneriaceae), a new species from eastern Guangxi, China

**DOI:** 10.3897/phytokeys.197.83089

**Published:** 2022-05-23

**Authors:** Chi Xiong, Wei-Chuen Chou, Yi Huang, Fang Wen

**Affiliations:** 1 Guangxi Key Laboratory of Plant Conservation and Restoration Ecology in Karst Terrain, Guangxi Institute of Botany, Guangxi Zhuang Autonomous Region and Chinese Academy of Sciences, CN-541006, Guilin, China Guangxi Institute of Botany Guilin China; 2 National Gesneriaceae Germplasm Resources Bank of GXIB, Gesneriad Committee of China Wild Plant Conservation Association, Gesneriad Conservation Center of China (GCCC), Guangxi Institute of Botany, Guilin Botanical Garden, Guangxi Zhuang Autonomous Region and Chinese Academy of Sciences, CN-541006, Guilin, China Guangxi Institute of Botany Guilin China

**Keywords:** Flora of Guangxi, Gesneriaceae, new taxon, *Primulinayangshuoensis*, taxonomy

## Abstract

*Primulinanana* C.Xiong, W.C.Chou & F.Wen, a new species of Gesneriaceae from limestone areas of Guangxi, China, is described and illustrated here. It morphologically resembles *P.yangshuoensis* Y.G.Wei & F.Wen in papillose leaf surface, but can be easily distinguished from the latter by noting a combination of characteristics, especially in its leaf blades, leaf blade indumentum characteristic, calyx lobes, corolla and the disc. We found only one population at the type locality, about 200 mature individuals. According to the IUCN Red List Categories and Criteria (Version 3.1), the new species is provisionally assessed as Critically Endangered (CR).

## ﻿Introduction

The genus *Primulinas.l.* was redefined in 2011, comprising *Chiritopsis* W.T.Wang, *Wentsaiboea* D.Fang & D.H.Qin (except *W.tiandengensis* Yan Liu & B. Pan) and the large number of species described in Chiritasect.Gibbosaccus C.B.Clarke (Wang et al. 2011; Weber et al. 2011). This genus now exhibits the most diversity in the Chinese Gesneriaceae, including approximately123 species and eight varieties of *Primulinas.l.* after the revision (Wang et al. 2011; Weber et al. 2011). An acceleration of *Primulina* species discovery has been seen over the last decade, with an average of about 10 new species per year. As of February 2022, there were 221 species (excluding infraspecific taxa) (GRC 2022) in this genus. China is the centre of diversity for *Primulina* with at least 204 species and 15 varieties occurring there at present (Wen et al. 2022), especially in limestone areas (Wei 2018; Wen et al. 2019; Ge et al. 2020; Liu et al. 2020; Xin et al. 2020a, b, c, 2021; Zhang et al. 2021). The tropical and subtropical karst limestone mountainous areas of Guangxi, China, are the centres of species diversity and differentiation of this genus (Li et al. 2019).

In October 2021, Y. Huang, a Gesneriaceae enthusiast from Guangxi, found this unknown plant in the wild. One of the authors, W.C. Chou, went to the type locality and collected specimens. Some of the living plants were introduced and cultivated at the Gesneriad Conservation Center of China (GCCC) and the National Gesneriaceae Germplasm Bank for further research. Comparison of the live plants with the type specimens and protologues of all known species of *Primulina* led to the determination that these specimens neither fit the existing protologues nor conform to the type specimens of these species. Nevertheless, the leaves’ tiny shape and texture make them very particular and most similar to *P.yangshuoensis* Y.G.Wei & F.Wen (Wen et al. 2012). However, a combination of characteristics easily distinguished it from other species, especially in its leaf blades, leaf blade indumentum, calyx lobes, corolla and the disc characters. We confirmed that it represents a new species of *Primulina* and describe it here.

## ﻿Taxonomic treatment

### ﻿ Primulina nana

Taxon classificationPlantaeLamialesGesneriaceae

C.Xiong, W.C.Chou & F.Wen sp. nov.

D7021A94-786B-54D0-8733-AE748FFACFD0

urn:lsid:ipni.org:names:77298577-1

Figs 1 , 2A1–F1

#### Diagnosis.

The new species resembles *Primulinayangshuoensis* (Fig. 2A2–F2) in papillose leaf surface, namely numerous single pubescent hair on papilla on surface, but can be easily distinguished from the latter by its leaf blade elliptic to ovate, 1.6–1.8 × 1.1–1.3 cm (vs. broadly ovate-round, subround or round, 3.5–6.5 × 2–4.5 cm); leaf nearly erect semi-transparent papillose-puberulent and white pubescent (vs. densely erect semi-transparent and white multicellular papillose-hispid); peduncle 3–5 cm long (vs. 8–9 cm); calyx lobes with one serration (vs. entire); corolla ca. 1.5 cm long, tube tubular (vs. 2–3 cm, broadly infundibuliform); disc ca. 0.6 mm high (vs. ca. 1 mm). Detailed morphological comparisons with *P.yangshuoensis* are provided in Table 1.

**Figure 1.**
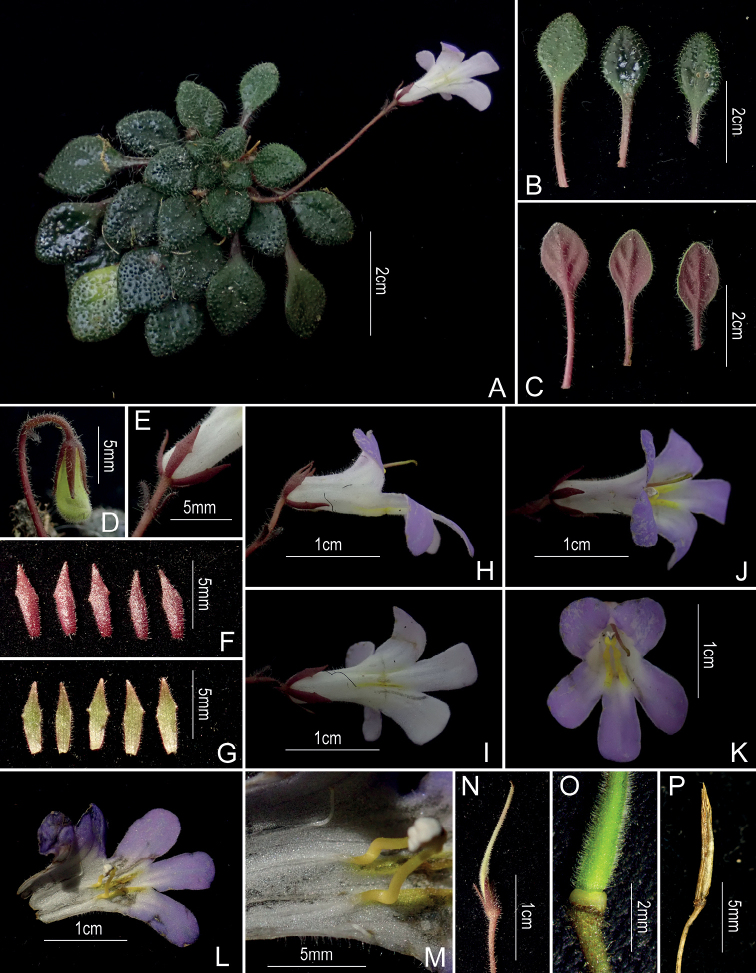
*Primulinanana* sp. nov. **A** habit **B** adaxial side of leaves **C** abaxial side of leaves **D** alabastrum **E** bracts and calyx **F** abaxial side of calyx lobes **G** adaxial side of calyx lobes **H** lateral view of a flower **I** ventral view of a flower **J** top view of a flower **K** front view of a flower s howing the internal structure **L** opened corolla **M** stamens and staminodes **N** pistil with calyx **O** disc and ovary **P** capsule. Photographs by Chi Xiong.

**Table 1.** Detailed comparison of *Primulinanana* and its relative *P.yangshuoensis*.

Characters	*P.nana*	*P.yangshuoensis*
Leaf-blades	elliptic to ovate, 1.6–1.8 × 1.1–1.3 cm	broadly ovate-round, subround or round, 3.5–6.5 × 2–4.5 cm
Leaf indumentum	densely nearly erect semitransparent papillose-puberulent on adaxial surface, 1–2 mm long, abaxially purple, densely white pubescent, 1–2 mm long	densely erect semitransparent or white multicellular papillose-hispid on both surfaces, 0.8–1 cm long on the adaxial surface, 4–5 mm long on the abaxial surface
Peduncle	3–5 cm long	8–9 cm long
Bracts	oblong to linear, 2.5–3 × ca.1 mm	lanceolate or subulate, 1–2 × 0.3–0.5 mm
Calyx lobes	Usually, one serrate at the middle	entire
Corolla	ca.1.5 cm long, tube tubular and gradually narrow to the bottom	2–3 cm long, tube broadly infundibuliform
Disc	ca. 0.6 mm high	ca. 1 mm high

#### Type.

China. Guangxi Zhuangzu Autonomous Region: Wuzhou City, Mengshan County, Xinxu Town, 24°19'N, 110°22'E, altitude ca. 530 m, November 26, 2021, *Chou Wei-Chuen & Huang Yi CWC211126-01* (IBK!)

#### Description.

Herbs perennial, acaulescent, rhizome subterete, ca. 1.5 cm long, 4–6 mm in diameter, Leaves 14–23, all basal, petiolate; petiole 1–3 cm long, ca. 1.5 mm in diameter, purple, densely pubescent; leaf blade elliptic to ovate, 1.6–1.8 × 1.1–1.3 cm, leathery, adaxially dark green to purplish-green, nearly erect semi-transparent papillose-puberulent on adaxial surface, 1–2 mm long, abaxially purple, white pubescent, 1–2 mm long, base broadly cuneate, margin entire, apex acute to obtuse, lateral veins inconspicuous, 2–3 on each side. Cymes 2–4, axillary; usually simple, peduncle 3–5 cm long, ca. 0.8 mm in diameter, pubescent;bracts 2, opposite, oblong to linear, 2.5–3 × ca. 1 mm, pubescent on both surfaces, margin entire, apex obtuse. Calyx 5-parted to base, lobes lanceolate, ca. 5 × 1–1.5 mm, nearly equal, outside purple, densely pubescent, inside yellow-green, nearly glabrous, usually with one serrate on the middle of calyx lobe, apex acute. Corolla purple, throat with two yellow stripes inside, ca. 1.5 cm long, outside glandular and eglandular puberulent, inside glabrous, tube tubular and gradually narrowing to the bottom, ca. 1 cm long, orifice 5–6 mm in diameter; limb distinctly 2-lipped, adaxial lip 2-parted to the middle, lobes oblong or round, apex round, 3–4 × 5–6 mm, abaxial lip 3-parted to near the base, lobes oblong, apex round, 6–8 × 4–5 mm. Stamens 2,adnate ca. 8 mm above the corolla base; filaments yellow, ca. 5 mm long, geniculate near the base, glabrous, anthers reniform, slightly constricted at middle, 1.5–2 mm long; staminodes 3, lateral ones linear, glabrous, ca. 3 mm long, apex capitate, glabrous, adnate to ca. 7 mm above the corolla tube base, the central one inconspicuous, adnate near the corolla tube base. Disc annular, ca. 0.6 mm high, margin repand, glabrous. Pistil 1.4–1.6 cm long, ovary 5–6 mm long, ca. 1 mm in diameter, glandular-pubescent; style ca. 1 cm long, 0.6 mm in diameter, glandular-pubescent; stigma obtrapeziform, ca. 1 mm long. Fruit linear, longitudinally dehiscent, 8–9 mm long, ca. 1.5 mm in diameter.

**Figure 2.**
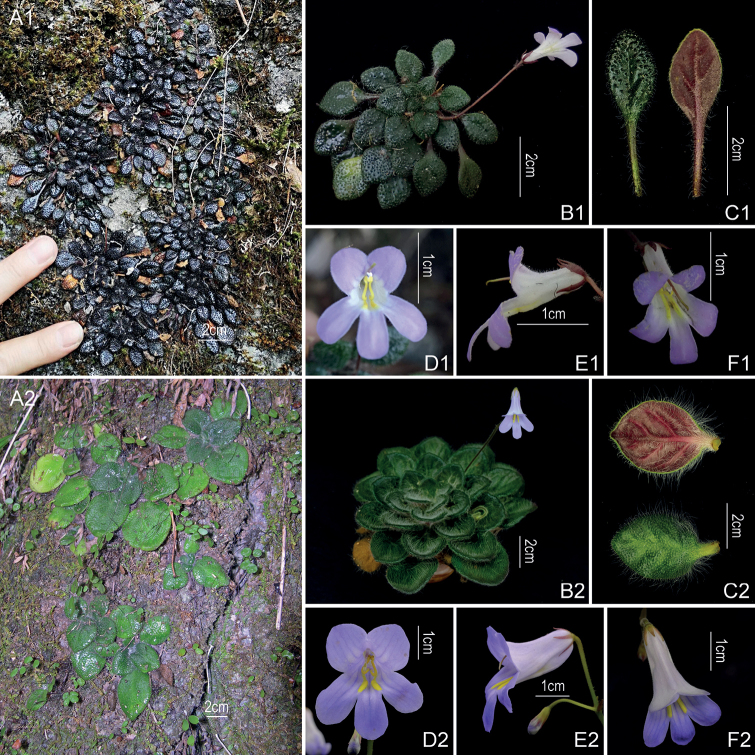
Morphological comparison of *Primulinanana* sp. nov. (1) with *P.yangshuoensis* (2) **A** habitat **B** habit **C** leaf blades **D** front view of flowers **E** lateral view of flowers **F** top view of flowers. **A1** and **D1** photographed by Wei-Chuen Chou; **B1–C1, E1–F1, C2** photographs by Chi Xiong; **A2–B2** and **D2–F2** photographed by Fang Wen.

#### Phenology.

Flowering in November, fruiting from December to the following January.

#### Etymology.

The specific epithet ‘*nana*’ is derived from the dwarf plants of the new species compared with that of most other species of *Primulina*.

#### Vernacular name.

玲珑报春苣苔 (Chinese name); Líng Lóng Bào Chūn Jù Tái (Chinese pronunciation).

#### Distribution and habitat.

*Primulinanana* is only known from the type locality, Xinxu Town, Mengshan County, Wuzhou City, Guangxi, China. It grows on moist, shady limestone rock surfaces, at an elevation of ca. 530 m.

#### Conservation status.

*Primulinanana* is only known from one population of about 200 mature individuals at the type locality. This population has been reduced by 90% from when it was originally found. The EOO and AOO of the new species are about 0.2 km^2^ and 25 m^2^, respectively. Its beautiful flowers, thickened woody rhizomes and shapely leaves, led to over-harvesting by locals, who sold it as an ornamental plant. Thus, following the IUCN Red List Categories and Criteria (IUCN 2019), it is temporarily assessed as Critically Endangered [CR B1ab (iii, v) + B2ab (iii, v)].

#### Notes.

The plant size of *Primulinanana* is dwarf and leaf blade length is less than 2 cm, but length of flowers is about 1.5 cm and the proportion of flowers and leaves is unusual in this genus. These characters differ from other *Primulina* species and can be clearly distinguished from *P.yangshuoensis* in morphological characters (Table 1).

## Supplementary Material

XML Treatment for Primulina nana
